# RNA-Seq of the Nucleolus Reveals Abundant SNORD44-Derived Small RNAs

**DOI:** 10.1371/journal.pone.0107519

**Published:** 2014-09-09

**Authors:** Baoyan Bai, Srinivasan Yegnasubramanian, Sarah J. Wheelan, Marikki Laiho

**Affiliations:** 1 Department of Radiation Oncology and Molecular Radiation Sciences, Johns Hopkins University School of Medicine, Baltimore, Maryland, United States of America; 2 Sidney Kimmel Comprehensive Cancer Center, Johns Hopkins University School of Medicine, Baltimore, Maryland, United States of America; National Institute of Health – National Cancer Institute, United States of America

## Abstract

Small non-coding RNAs represent RNA species that are not translated to proteins, but which have diverse and broad functional activities in physiological and pathophysiological states. The knowledge of these small RNAs is rapidly expanding in part through the use of massive parallel (deep) sequencing efforts. We present here the first deep sequencing of small RNomes in subcellular compartments with particular emphasis on small RNAs (sRNA) associated with the nucleolus. The vast majority of the cellular, cytoplasmic and nuclear sRNAs were identified as miRNAs. In contrast, the nucleolar sRNAs had a unique size distribution consisting of 19–20 and 25 nt RNAs, which were predominantly composed of small snoRNA-derived box C/D RNAs (termed as sdRNA). Sequences from 47 sdRNAs were identified, which mapped to both 5′ and 3′ ends of the snoRNAs, and retained conserved box C or D motifs. SdRNA reads mapping to SNORD44 comprised 74% of all nucleolar sdRNAs, and were confirmed by Northern blotting as comprising both 20 and 25 nt RNAs. A novel 120 nt SNORD44 form was also identified. The expression of the SNORD44 sdRNA and 120 nt form was independent of Dicer/Drosha–mediated processing pathways but was dependent on the box C/D snoRNP proteins/sno-ribonucleoproteins fibrillarin and NOP58. The 120 nt SNORD44-derived RNA bound to fibrillarin suggesting that C/D sno-ribonucleoproteins are involved in regulating the stability or processing of SNORD44. This study reveals sRNA cell-compartment specific expression and the distinctive unique composition of the nucleolar sRNAs.

## Introduction

The nucleolus contains a rich presentation of RNAs. Ribosomal (r) RNA biosynthesis comprises the main metabolic activity of the nucleolus. rRNA transcription is driven by a highly active dedicated polymerase, RNA polymerase I (Pol I), that transcribes rDNA genes to 47S precursor rRNA. The 47S precursor is processed to the mature 28S, 18S and 5.8S RNAs by multiple steps that require the activity of proteins and enzymes for proper cleavage, modification and folding of the rRNAs. The modification and folding of rRNAs is supported by numerous small nucleolar RNAs (snoRNA) that are essential in guiding the proper positioning of rRNAs in large ribonucleoprotein (RNP) complexes [1]–[3]. The mature rRNAs are assembled to ribosomal 60S and 40S particles and translocated to the nucleus for further maturation [4]. This key metabolic activity, ribosome biogenesis, coordinates the assembly of the nucleolus into distinct subnucleolar domains that build around individual transcription and processing sites.

Human snoRNAs are highly evolutionarily conserved 60–300 nt long non-coding RNAs, and typically arise from intronic sequences [5], [6]. The two main classes of snoRNAs consist of the box C/D snoRNAs that contain box C (RUGAUGA) and D (CUGA) motifs, and the H/ACA snoRNAs that share a conserved box H (AnAnnA) and ACA motifs [1], [7]. The box C/D and H/ACA snoRNAs assemble with distinct protein complexes, and govern distinct functions. Box C/D snoRNAs act as guides for 2′-*O*- methylation of rRNA sequences, and assemble in a dimeric asymmetric complex with the proteins 15.5K, NOP56, NOP58 and fibrillarin (FBL) [8]–[11]. FBL binds the guide-substrate RNA duplex and executes substrate RNA methylation. The H/ACA snoRNAs mediate their substrate RNA pseudouridylation through the catalytic activity of dyskerin. While the major function of snoRNAs is the modification of rRNAs, they also mediate methylation and pseudouridylation of snRNAs, tRNAs and some mRNAs [7], [11].

In addition to snoRNAs, multiple RNA species have defined tasks in the nucleolus, or visit there transiently for modification or processing. In *Xenopus laevis*, several small nuclear RNAs (snRNAs) transiently locate to the nucleolus [12], [13]. The RNase P RNA assists in the 5′ processing of tRNA in the nucleolus [14]. At least one microRNA (miRNA) has been reported in the nucleolus of rat myoblasts [15], [16], and several nucleolar miRNAs were demonstrated in HeLa cells in a recent study [17]. Besides rRNA and ribosome biogenesis, a number of other functions that involve RNP assemblies have been associated with the nucleolus. The signal recognition particle proteins together with the cognate 7S RNA undergo assembly in the nucleolus [18], [19]. The functionality of the telomerase complex is modulated by nucleolar activities [20], [21], possibly providing a link between the nucleolus in the control of aging [21].

Several reports have recently identified small (s) (18–22 nt) RNA derivatives of the snoRNAs, termed as snoRNA-derived RNAs (sdRNA) [22]–[27]. These data have arisen from sRNA deep sequencing studies or bioinformatics analyses of deep sequencing datasets, and have shown that several sdRNAs have miRNA-like properties, or regulate alternative mRNA splicing [22], [26]–[28]. Sequencing of small RNA co-precipitating with Ago2 protein by photoreactive nucleotide-enhanced crosslinking and immunoprecipitation (PAR-CLIP) however showed that sRNAs derived from snoRNA are less likely to be incorporated into Ago 2 complex suggesting sdRNAs do not share this miRNA-like property [29]. Conversely, many human miRNA precursors contain box C/D or box H/ACA sequences, bind cognate proteins FBL and dyskerin, and are predicted to share snoRNA fold structures [30], [31]. However, alternative formation of RNP complexes with the sdRNAs has been noted as well. SNORD115 sdRNA associates with hnRNP proteins but lacks binding with FBL and NOP58 [28].

Given the rich representation of nucleolar RNAs, especially rRNA and snoRNAs, and reports of abundant expression of sRNA derivatives, we wanted to address the subcellular distribution of the sRNAs. We wanted to resolve whether sdRNAs are detected in the nucleolus and asked whether their expression or localization is affected by pathways involved in sRNA processing or by rRNA transcription. We therefore used massively parallel small RNA sequencing (sRNA-seq) to investigate in an unbiased manner the expression of <40 nt RNAs isolated from nuclear, cytoplasmic, nucleolar or total cellular fractions. We show here that the small RNome of the nucleolus has many unique features. These include its distinct size distribution and extremely high representation of box C/D sdRNAs, especially that of SNORD44. The majority of the nucleolus-associated sdRNAs were also detected in the nuclear fraction. In contrast, only very few sdRNAs were detected in the cytoplasm. The nucleolar expression of SNORD44 sdRNA was further validated by Northern hybridization and shown to depend on the expression of FBL and NOP58, but not on the canonical miRNA-processing pathway. A novel 120 nt form of SNORD44 was identified. This study provides a comprehensive analysis of the subcellular distribution of sRNAs in HeLa cells.

## Materials and Methods

### Cell Lines and Reagents

HeLa cervical adenocarcinoma cells (CCL-2, ATCC) and HCT116 cells (wild type and DICER−/−) were maintained in DMEM supplemented with 10% FCS. HCT116 and DICER−/− cells were a kind gift of Dr. V. Velculescu (Johns Hopkins University, Baltimore, MD). All cell culture reagents were obtained from Invitrogen. Actinomycin D (ActD, A1410) and Leptomycin B (L2913) were from Sigma-Aldrich.

### Fractionation of Cells

Subcellular fractionation was carried out essentially as in ref. [32]. 1.8×10^8^ cells were used for isolation of the nucleolar fraction. Briefly, the cytoplasmic and nuclear compartments were separated using hypotonic lysis, and the nuclear fraction was further subjected to sucrose gradient centrifugation to isolate nucleoli. The nuclear fraction contained also the nucleoli. In addition, RNA was extracted from whole cells (denoted here as cellular RNA).

### RNA Isolation and Generation of cDNA Libraries

**Figure 1:**
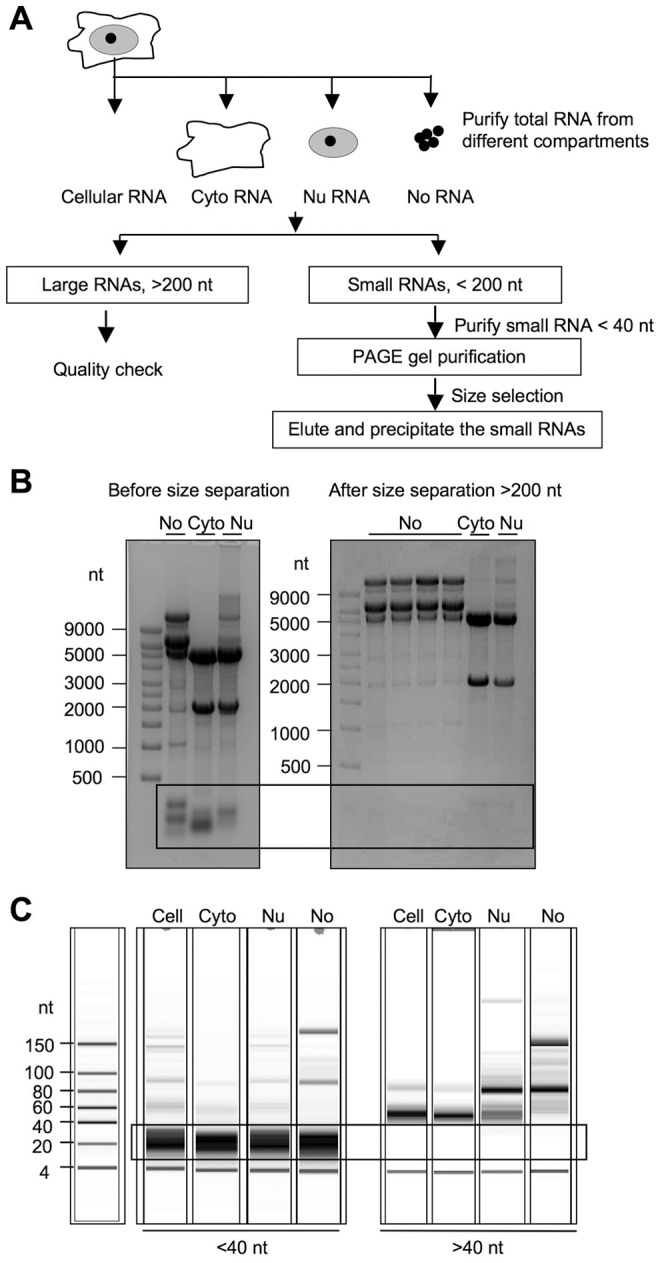
sRNA-seq strategy and preparation of small RNA libraries. *A* Subcellular fractionation and RNA purification scheme. Cyto, cytoplasmic; Nu, nuclear; No, nucleolar. *B* RNA-PAGE analysis by 16% denaturing PAGE before (*left*) and after (*right*) purification of the <200 nt RNA fraction. Subcellular fractions are indicated at the top. *C* RNA profiles of the <40 nt (*left*) and >40 nt fractions (*right*) as analyzed by BioAnalyzer. Subcellular RNA fractions are indicated on *top*, RNA size markers to the *left*.

Total RNA was extracted from whole cells and the isolated cellular compartments using the TRIzol Reagent (15596-018, Invitrogen) according to manufacturer's instructions as outlined in Figure 1*A*. Approximately 400 µg total RNA from each compartment was used to prepare cDNA libraries. Briefly, small RNAs (less than 200 nt) were isolated from the total RNA samples using mirVana Isolation Kit (Ambion) and separated on denaturing 15% polyacrylamide gel (15% PAA, 19∶1 acrylamide/bis, 7 M urea) to purify RNAs in the approximate range of 10–40 nt. The sRNA size range was confirmed and the RNA was quantified using Bioanalyzer and the Agilent Small RNA kit (Agilent). cDNA libraries were constructed using the Ion Torrent RNA-seq kit v1 for small RNA libraries according to manufacturer's instructions, and their purity and concentration was confirmed using Bioanalyzer with the Agilent DNA kit. sRNA library sequencing was performed on a 314 chip on an Ion Torrent sequencing platform (Life Technologies, Invitrogen) at the Johns Hopkins Sidney Kimmel Comprehensive Cancer Center (SKCCC) Next Generation Sequencing Core. Raw data is deposited to GEO as GSE50057.

### RNA-seq and Data Analysis

All raw reads were automatically trimmed to remove adaptors and then aligned on human genome (hg19 GRCh37) and annotated using Torrent Suite 1.5 (TMAP) with default parameters. Only reads mapping to unique positions were considered. In addition, all reads were aligned with rDNA (U13369 and AL592188). Small and non-coding RNAs were classified according to the gene types. As indicated, analyses were conducted on reads represented at frequency of ≥10 for each sdRNA. Box C and D motifs were identified in the reads according to their consensus sequences (TGATGA and CTGA, respectively). One mismatch was tolerated for the box C motif and none for D motif. Guide RNA targets were identified according to snoRNA Orthological Gene Database (snOPY: http://snoopy.med.miyazaki-u.ac.jp/). SnoRNA secondary structure prediction was according to MFold (http://mfold.rna.albany.edu/?q=mfold).

### Northern Hybridization

Northern blotting was carried out according to ref. [33] with some modifications. Briefly, 20–25 µg RNA was resolved on denaturing polyacrylamide gels in 1XTBE, transferred onto nylon membranes (Roche Diagnostics) using semi-dry electroblotting (Bio-Rad), and immobilized by UV irradiation at 120 mJ/cm^2^ (Stratagene Crosslinker). The membrane was pre-hybridized with hybridization buffer at 37°C for 1 h, and then hybridized overnight at 37°C with specific oligodeoxynucleotides. The probes were labeled with digoxigenin using the DIG oligonucleotide 3′END labeling kit (Roche) and detected by the DIG Nucleic Acid Detection Kit (Roche). The following probes were used for SNORD44: 5′ upstream, GCTGCATTTACAAACTTTCTT; 5′, AGTTAGAGCTAATTAAGACCT; 3′, AGCTAATTAAGACCTTCATGT; 3′downstream, TGCCAAAGCTAACAAATGCCT; and for hY1, 5′-AAGGGGGGAAAGAGTAGAACA-3′. The Northern blotting signals were quantified and normalized to mature SNORD44. All quantifications were conducted on short exposures to avoid signal saturation.

### Immunofluorescence

HeLa cells grown on glass coverslips were fixed with 3.7% paraformadehyde and permeabilized with 0.5% NP40. The following primary antibodies were used: Rabbit anti-FBL (ab5821, Abcam), rabbit anti-Drosha (ab12286, Abcam) and rabbit anti-DGCR8 (ab36865, Abcam). Antibodies were detected with secondary antibodies conjugated to Alexa 488 or 594 (Molecular Probes, Invitrogen) and nuclei were counterstained with Hoechst 33258. Images were captured using Axioplan2 fluorescence microscope (Zeiss) equipped with AxioCam HRc CCD-camera and AxioVision 4.5 software using EC Plan-Neofluar 40x/0.75 objective (Zeiss). Image quantification was carried out according to refs. [34], [35].

### Nascent rRNA Synthesis

Cells were incubated with 1 mM 5-fluorouridine (FU) (Sigma-Aldrich) using hypotonic shift and fixed with ice-cold methanol and acetone according to ref. [36]. FU was detected using anti-5-BrdU antibody (Sigma-Aldrich) and Alexa 594 conjugate. DNA was counterstained with DAPI.

### *In Situ* Hybridization

Cells grown on coverslips were fixed in 4% PFA for 10 min. The cells were washed three times in PBS and permeabilized with 0.5% Triton X-100 for 10 min. The cells were then rehydrated in PBS for 10 min and pre-hybridized in 40% formamide in 2X SS (sodium chloride-sodium phosphate-EDTA buffer) for 20 min. DNA probes were diluted in hybridization buffer (50% formamide, 5X SSC, 250 µg/ml *E. coli* tRNA, 500 µg/ml salmon sperm DNA, 2% Roche blocking reagent, 0.02% Tween-20, 0.05% CHAPS in DEPC treated water) and incubated at 37°C for 5 h. Coverslips were washed with 5X SSC for 15 min at 37°C, twice for 35 min each at 37°C in 0.2X SSC and then once in PBS for 15 min at RT. Coverslips were blocked in 4% sheep serum and 3% BSA in PBS for 1 h and incubated in mouse anti-digoxigenin solution at 37°C for 1 h. Digoxigenin was detected with secondary antibodies conjugated to Alexa 488 or 594 (Molecular Probes, Invitrogen) and nuclei were counterstained with Hoechst 33258. The Dig-labeled *in situ* oligonucleotide probes for U3 snoRNA was as in ref. [37], SNORD44 5′-AGTTAGAGCTAATTAAGACCT and scrambled SNORD44 5′-AGTTAGAGTTATTCAAGACCT.

### RNAi

HeLa cells were transfected using Lipofectamine RNAiMAX (Invitrogen) with siRNAs (10 nM) at the time of plating and incubated for 48 or 72 hours. The following siRNAs were used: control siRNA and RPA194 siRNA (si403) were from Ambion [36], and Drosha [38] FBL and NOP56 [39] were synthesized by Integrated DNA Technologies.

### RNA Immunoprecipitation

RNA immunoprecipitation was conducted as previously described [40]. Purified nuclei were lysed in NP40 buffer to solubilize proteins. The nuclear isolate used for the precipitation contained approximately 150 µg total nuclear RNA. The FBL complex was immunoprecipitated using 10 µg rabbit anti-FBL antibody (Abcam) or rabbit IgG and collected using 50 µl Dyna Beads (Invitrogen). Immunoprecipitated RNA was isolated using TRIzol according to manufacturer's instruction and quantified. The samples were analyzed on 15% acrylamide/urea PAGE and subjected to Northern blotting analysis.

## Results

### RNA-seq of sRNA Libraries from Cellular Subcompartments

To investigate the sRNA subcellular distribution we separated cytoplasmic, nuclear and nucleolar fractions of HeLa cells according to established protocols (Fig. 1*A*) [32], [41]. Small RNAs (<40 nt) were further size-selected using MirVANA kit from the <200 nt fraction, and gel-purified (Fig. 1*B*). The nucleolar fraction was assessed for purity based on distinct protein and RNA expression profiles shown in Figure 1*B* and in ref. [32], [41]. The RNAs were size fractionated to fractions over and less than 200 nt (Fig. 1*B*). The purity and concentrations of <40 nt RNAs were analyzed using Bioanalyzer. As shown in Figure 1*C*, the 15–40 nt region was highly enriched and equally represented in all subcellular RNA fractions. cDNA libraries were prepared from each <40 nt RNA isolates.

The cDNA libraries were sequenced on an Ion Torrent deep sequencing platform using a 314 chip. Approximately 400,000 raw reads were recovered from each library, except from the nucleolus, which yielded 120,000 raw reads (Table 1). Of all raw reads, 60–70% aligned to the human genome sequence (hg19 GRCh37), and the lowest alignment percentage was obtained for the nucleolar library (Table 1). Among the non-nucleolar fractions, 50% of all aligned reads mapped to annotated sRNA loci. These included miRNAs, snoRNAs, snRNAs, and Y RNAs, vault RNAs and Linc RNAs. Examples of selected sRNA reads in the subcellular compartments are shown in Fig. S1. However, only 25% of the sRNA reads in the nucleolar library mapped to annotated loci indicating that nucleolar sRNA reads are less well annotated (Table 1). In addition, 8% nucleolar sRNA reads aligned with rDNA, especially at the 5′ETS region (Table 1).

**Table 1:** RNA-seq read alignment.

	Raw reads	Aligned reads	%Aligned	Aligned sRNA reads	%Aligned sRNA	rRNA reads	%rRNA
Cellular	352,664	252,832	71.7	190,878	54.1	796	0.3
Cytoplasm	395,975	275,309	69.5	198,631	50.2	788	0.3
Nucleus	416,199	283,762	68.2	204,996	49.3	2,070	0.7
Nucleolus	122,016	70,439	57.7	31,061	25.5	5,604	8.0

sRNA: miRNA, snoRNA, snRNA, Y RNA, Vault RNA, LincRNA.

The vast majority of the cellular, cytoplasmic and nuclear sRNA reads aligned to miRNA loci (98–99%), whereas 93% of the nucleolar sRNA reads aligned to snoRNA loci (Table 2). However, miRNAs were also represented in the nucleolar library by 6.8% of the annotated loci (Table 2). The data on miRNAs detectable in the nucleolar fraction are separately presented in ref. [34].

**Table 2:** Small RNA read distribution.

	sRNA reads	miRNA reads	%miRNA	snoRNA reads	%snoRNA	snRNA reads	%snRNA	Misc RNA	%Misc RNA
Cellular	190,878	188,913	99.0	922	0.48	28	0.01	1,015	0.53
Cytoplasm	198,631	197,780	99.6	125	0.06	25	0.01	701	0.35
Nucleus	204,996	200,095	97.6	4,245	2.07	110	0.05	540	0.26
Nucleolus	31,061	2,108	6.8	28,865	92.93	44	0.14	44	0.14

sRNAs: miRNAs, snoRNAs, snRNAs.

misc RNAs: Y RNAs, vault RNAs, lincRNAs.

**Figure 2:**
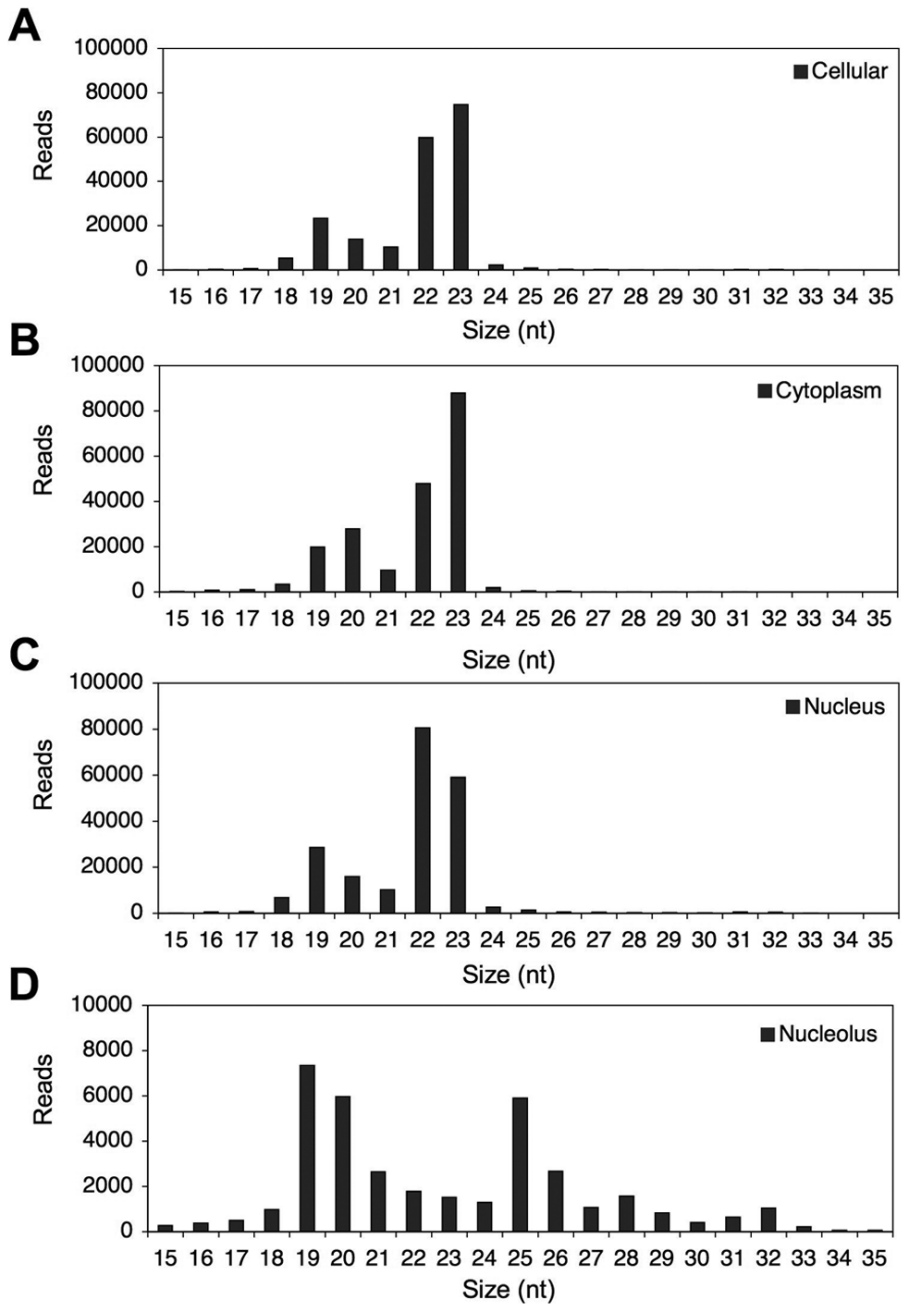
Size-distribution of the annotated small RNA sequence reads in the subcellular compartments. *A* Cellular RNA reads. *B* Cytoplasmic RNA reads. *C* Nuclear RNA reads. *D* Nucleolar RNA reads.

The size distribution of the sequence reads aligning to annotated loci differed among the cellular compartments. Sequence reads from the cellular, cytoplasmic and nuclear fractions were highly abundant in 22–23 nt size range, and reflected the preponderance of miRNA annotation for these fractions (Fig. 2*A*–*D*). In contrast, the nucleolar sRNAs were predominantly represented by 19–20 nt and 25 nt reads (Fig. 2*D*). Thus, while sRNAs were abundantly present in all subcellular compartments, the nucleolus-associated small RNome displayed certain unique characteristics. In this report, we focused on the expression and size-distribution of sRNAs deriving from the snoRNA loci.

### Nucleolar sRNA Reads are Predominantly Derived from snoRNA Loci

**Figure 3:**
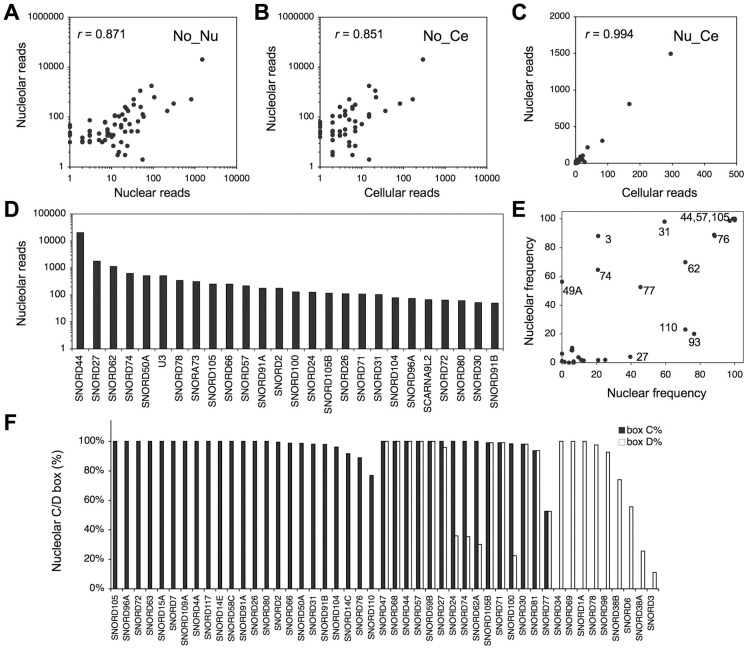
Distribution of sdRNAs in the cellular subfractions. *A–C* Scatter plots of sdRNA reads in the different cellular compartments. Note the differential scaling of the axes. Pearson correlation coefficiences are indicated (*r*). *A* Nucleolar (No) *vs*. nuclear (Nu) sdRNA reads. *B* Nucleolar (No) *vs*. whole cell (Ce) sdRNA reads. *C* Nuclear (Nu) *vs.* whole cell (Ce) sdRNA reads. *D* Nucleolar sdRNA reads (reads ≥50 are shown). *E* Scatter plot of frequencies of nucleolar and nuclear 5′ reads present at ≥10 reads in both libraries. Selected SNORDs are identified by their numbering. *F* C/C' and D/D' box frequencies of nucleolar sdRNA reads.

SdRNAs were highly represented in the nucleolus (28,865 reads) but very few were detected in the cytoplasm (125 reads)(Table 2). To ask whether the nucleolar read numbers reflect the frequency of reads in other cellular compartments we first plotted and compared their read frequencies. The frequency distribution of the nuclear and whole cell sdRNA reads differed from that of the nucleolar reads (Fig. 3*A and B*). However, the sdRNA reads in the nuclear and cellular fractions showed a strong correlation (*r* ^2^ = 0.994, Pearson's correlation coefficiency) (Fig. 3*C*).

Further analysis of the 68 snoRNA loci represented by more than 10 reads in any given fraction showed that 63 mapped to box C/D and 5 to box H/ACA snoRNA loci, respectively (Table S1) showing that box C/D snoRNA-derived reads were highly frequent. On the other hand, sdRNAs of only three Cajal body snoRNAs (SCARNA6, SCARNA15, SCARNA9L2) were represented in the subcellular libraries. Of these, SCARNA15 was not detected in the nucleolus at all, and had the highest read number of all cytoplasmic sdRNAs (Table S1).

The most abundantly represented locus among the sdRNA reads in the nucleolar fraction was SNORD44 (RNU44) comprising 71.4% of all nucleolar sdRNAs (Fig. 3*D*). Notably, all of the *GAS5* intronic snoRNA loci (SNORD47, SNORD74, SNORD75, SNORD76, SNORD77, SNORD78, SNORD79, SNORD80 and SNORD81) were represented among the nucleolar reads. Reads deriving from other intronic snoRNA families, such as *SHG1* (SNORD22, SNORD25, SNORD26, SNORD27, SNORD28, SNORD29, SNORD30, SNORD31) and *NOL5A* (SNORA51, SNORD56, SNORD57, SNORD86, SNORD110) were also detected, suggesting that a number of mature snoRNAs from the same locus may be processed into sdRNAs (Table S1).

The positioning of the reads within the snoRNA loci showed a slight preference of the reads mapping to a position corresponding to the 5′ end of the mature snoRNA sequence (56.7% and 43.3% for 5′ and 3′end reads, respectively) (Table S1). To assess whether the tendency to retain either 5′ or 3′ reads was comparable between the subcellular compartments, we analyzed the relative frequency of the 5′ reads in the nuclear and nucleolar fractions. The analysis indicated that most sdRNA reads showed a similar frequency of reads mapping to the 5′ end of the snoRNA in both the nucleolar and nuclear fraction (*e.g.* SNORD44, SNORD105, SNORD57) (Fig. 3*E*). For other loci, like SNORD31 and U3, the percentage of reads mapping to the 5′ end was considerably higher in the nucleolar fraction (98% and 89%, respectively), as compared to the nuclear fraction (59% and 21%, respectively) (Fig. 3*E*). The data thus indicated that while there appeared to be retention of a specific 5′ or 3′ end reads, this varied between the subcellular compartments in a snoRNA-dependent manner. This also suggested that the preference to retain only one sdRNA sequence emanating from a snoRNA was not uniform.

Further analysis of box C and D prevalence among the nucleolar sdRNA reads indicated that 23 sdRNAs contained box C, 9 box D and 15 both box C and D motifs (Fig. 3*F*). The data shows abundant, but variable retention of these conserved motifs in the sdRNA reads. Of all nucleolar sdRNA reads, only 5% of the reads from 9 sdRNAs retained guide RNA sequences (not shown).

### SNORD44 is Present in the Nucleolus as Short (19–25 nt), Long (120 nt) and Mature Forms

**Figure 4:**
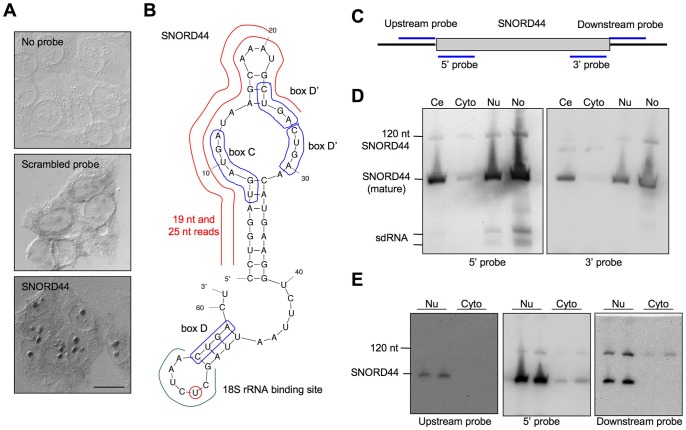
Analysis of SNORD44 sdRNAs. *A* SNORD44 *in situ* hybridization with the indicated probes. Differential interference contrast images. Scale bar 20 µm. *B* Predicted secondary structure of the mature SNORD44 (MFold). Positions of box C/C' and box D/D' and 18S RNA binding site are indicated. Red lines show 19 nt and 25 nt sdRNA read locations. *C* SNORD44 probes used for Northern hybridization. *D* Northern analysis of SNORD44 expression in the cell subcompartments using 5′ (*left*) or 3′ (*right*) SNORD44 probes. Ce, whole cell; Cyto, cytoplasmic, Nu, nuclear; No, nucleolar. *E* Northern analysis of SNORD44 expression in nuclear and cytoplasmic RNA fractions using the indicated probes. An equal amount of each RNA preparation (25 µg) was loaded in duplicate. Note that the SNORD44 signal in the hybridizations with the upstream and downstream probes is a bleed through of the earlier probing with the mature 5′ probe.

We then focused on the SNORD44 locus due to its high read frequency in the nucleolus. We first verified that SNORD44 is detectable in the nucleolus using *in situ* hybridization (Fig. 4*A*). Mature SNORD44 is a 61 nt transcript expressed from the intronic *GAS5* locus. sdRNA reads from this locus showed a distinct dual-peak length distribution of 19 and 25 nt, respectively, the 19 nt reads containing the 5′ box C and the 25 nt reads containing the 5′ box C and box D' (Fig. 4*B*). We used RNA preparations from the cellular compartments to analyze SNORD44 expression by Northern hybridization, employing probes targeting the mature form or flanking regions (Fig. 4*C*). As shown in Figure 4*D*, SNORD44 was highly abundant in the nucleolar fraction, and also detectable in the nucleus and whole cell fractions, but very low in the cytoplasm. This was in good correlation with the read frequencies observed by sRNA-seq. Also, consistent with sRNA-seq, two distinct 20–25 nt fragments were detectable in both the nucleolar and nuclear RNA fractions (Fig. 4*D*). Northern blotting using probes targeting the 5′ and 3′ ends of the mature SNORD44 confirmed that both small fragments derived exclusively from the 5′ end of the locus (Fig. 4*D*). The Northern hybridization further indicated the existence of an approximately 120 nt transcript (Fig. 4*D*). These size estimations were based on probing the filters with several sRNAs with known sizes (hY1, 112 nt; U3, 217 nt; U6, 106 nt; U11, 135 nt, and miR-21 22 nt) (not shown). Northern analysis of nuclear and cytoplasmic RNA samples using probes from the regions immediately up- or downstream of the mature SNORD44 suggested that the longer transcript represented a 3′extension of the mature SNORD44 (Fig. 4*E*). These findings confirmed the expression of nucleolar SNORD44 sdRNAs and revealed the expression of a longer SNORD44 transcript that we refer here to as 120 nt SNORD44.

### Neither Drosha nor Dicer are Involved in the Processing of sRNAs Derived from the SNORD44 Locus

**Figure 5:**
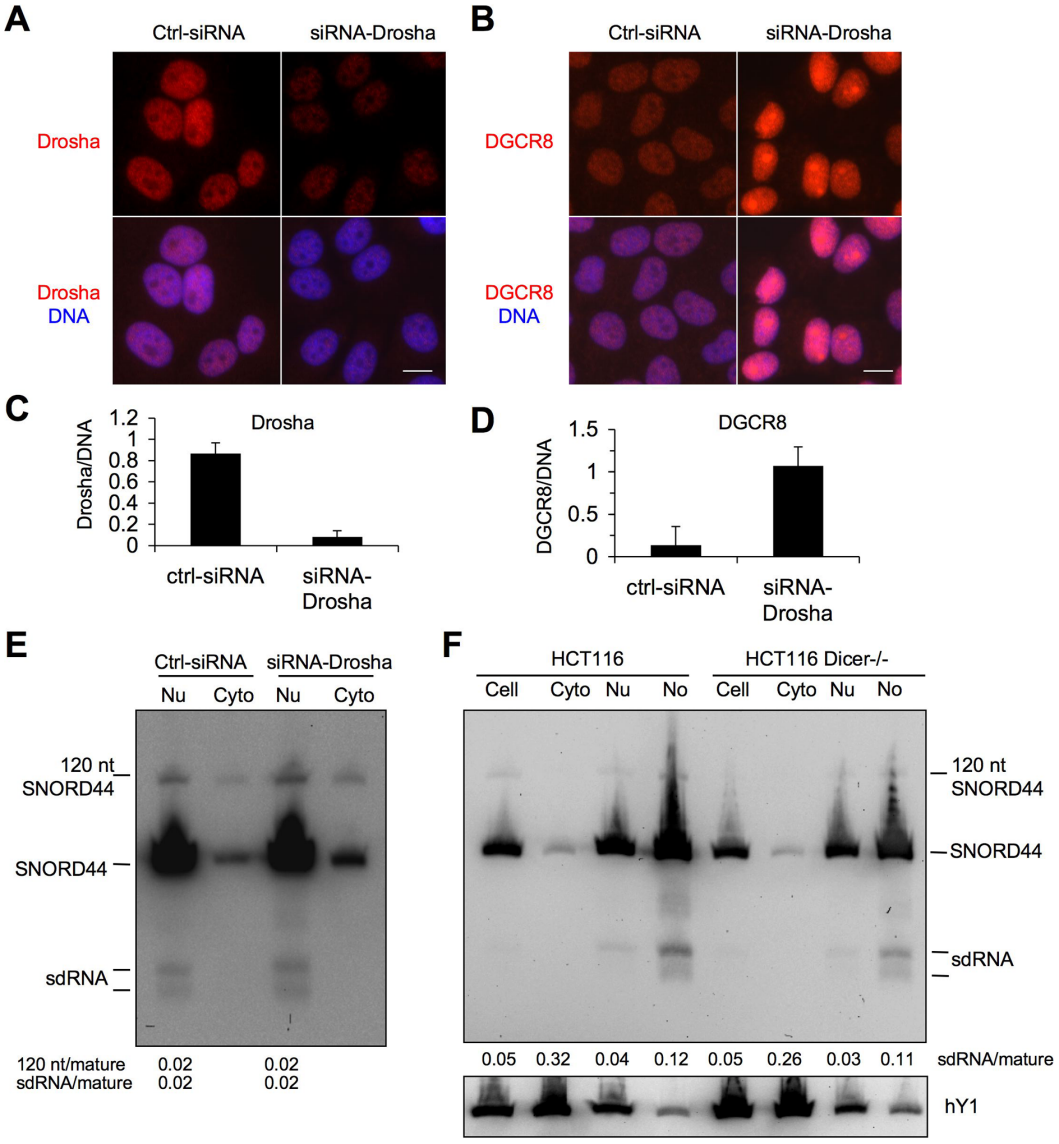
The canonical siRNA-processing pathway is not involved in the regulation of SNORD44 sdRNAs. *A–D* HeLa cells were transfected with control or Drosha-targeting siRNA and incubated for 48 hours. Cells were fixed and stained for Drosha (*A*) or DGCR8 (*B*), and counterstained for DNA. Merged images are shown. Scale bar, 10 µm. (*C, D*) Image quantifications for (*C*) Drosha and (*D*) DCGR8. Mean normalized fold intensity is shown. Error bars, SD. *E* Nuclear (containing nucleoli) and cytoplasmic fractions were prepared of the cells described in A, and RNA was isolated. Northern hybridization was conducted using the SNORD44 5′ probe. Signal intensities for 120 nt and sdRNAs were quantified and normalized against the mature SNORD44. *F* HCT116 and HCT116 Dicer −/− cells were subjected to subcellular fractionation, RNA was isolated and Northern hybridization was conducted using the SNORD44 5′ probe. Signal intensities for sdRNAs normalized to mature SNORD44 are provided below. hY1 probe was used as control.

Several snoRNAs are predicted to undergo processing to miRNA-sized RNA fragments, but their processing pathways are not known [42], [43]. Dicer has been implicated in the processing of H/ACA snoRNA [30] but not box C/D snoRNAs [44]. We hence explored the possibility that the miRNA pathway is involved in the processing of the SNORD44-derived fragments. To this end we first depleted Drosha by transfection of HeLa cells with Drosha-targeting siRNAs. Fluorescence microscopy and image quantification showed over 90% decrease of Drosha (Fig. 5*A and C*). As further evidence of Drosha functional inactivation, we observed a prominent increase in the expression of DGCR8, consistent with its previously shown negative regulation by Drosha (Fig. 5*B and D*) [38]. However, as assessed by Northern hybridization of the nuclear and cytoplasmic RNA fractions, depletion of Drosha did not affect the expression of either the SNORD44 sdRNAs or its 120 nt transcript (Fig. 5*E*). We next assessed the abundance of SNORD44 in the subcellular fractions of Dicer knock down cells [35]. There was no reduction of SNORD44 sdRNAs in the Dicer −/− HCT116 cells as compared to the parental Dicer-proficient cells (Fig. 5*F*). Conversely, others and we have shown that there is at least 2-fold decrease of miR-21 in the Dicer −/− HCT116 cells [34], [44]. The data indicate that the expression of the SNORD44 sdRNAs is independent of the canonical miRNA-processing pathway.

### The Abundance of SNORD44 sdRNA is Affected by Actinomycin D but not by Decreasing Pol I Transcription Rate

**Figure 6:**
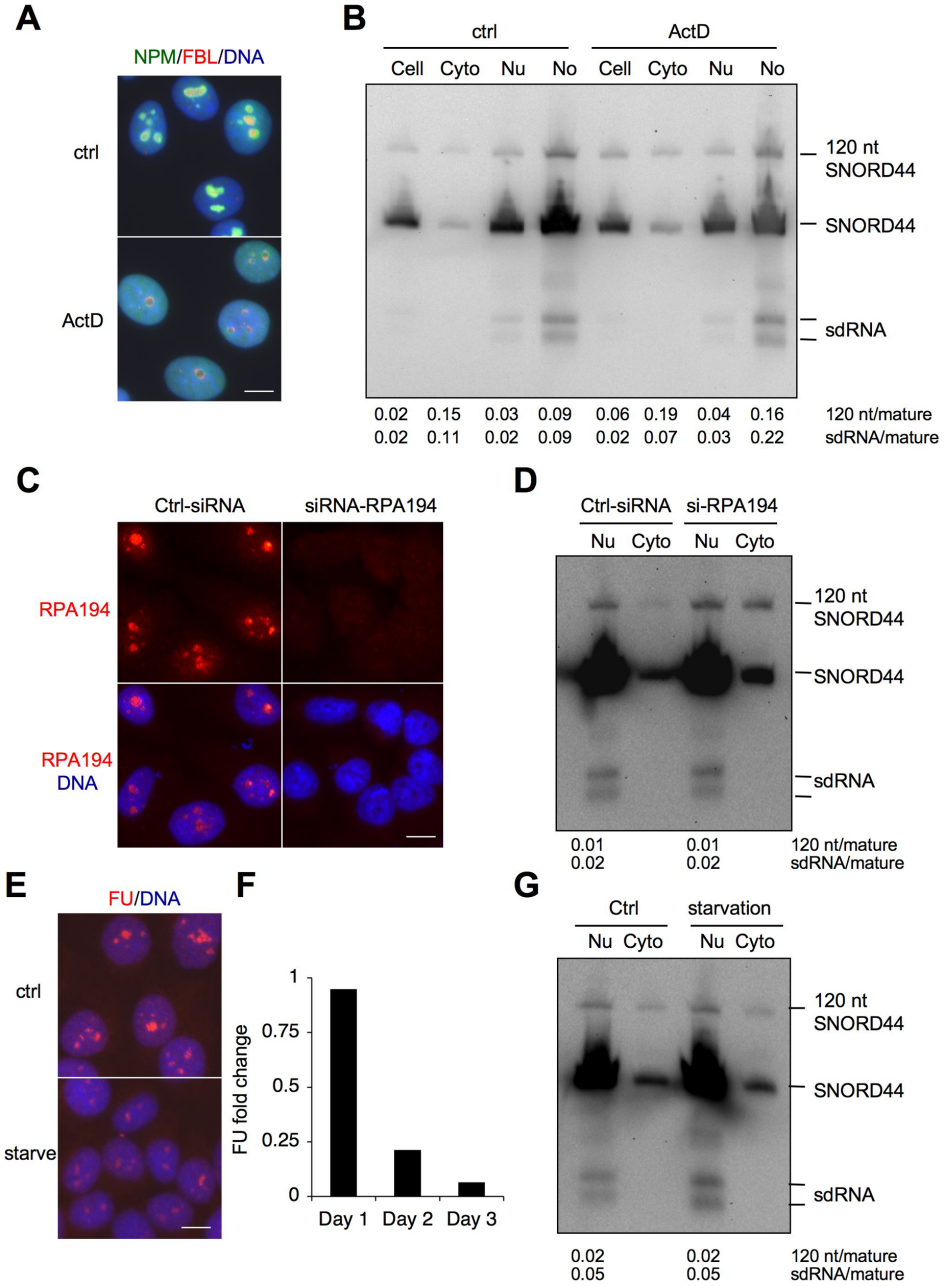
Effect of Pol I transcription blocks and transcription rate on SNORD44 and its sdRNA and long form. (*A, B*) HeLa cells were treated with Actinomycin D (Act D, 50 ng/ml) and incubated for 3 hours. *A* Cells were stained for NPM (*green*), FBL (*red*) and DNA (*blue*). Merged images are shown. Scale bar 10 µm. *B* Cells were subjected to subcellular fractionation, RNA was isolated and Northern hybridization was conducted using SNORD44 5′ probe. (*C and D*) HeLa cells were transfected with control or RPA194-targeting siRNA and incubated for 48 hours. *C* Cells were stained for RPA194 (*red*) and DNA (*blue*). Merged images are shown. Scale bar 10 µm. *D* Nuclear (Nu) and cytoplasmic (Cyto) fractions were prepared, and RNA was isolated. Northern hybridization was conducted using SNORD44 5′ probe. (*E–G*) HeLa cells were starved for 48 hours in serum-depleted medium. (*E and F*) Cells were incubated with 5-fluorouridine (FU) for 1 h, detected with 5-BrdU antibodies (*E*) and quantified (*F*). The decrease in FU incorporation over days 1–3 of starvation is shown. Scale bar 10 µm. *G* RNA was isolated and Northern hybridization was conducted using SNORD44 5′ probe. (*B, D, G*) Signal intensities for 120 nt and sdRNAs were quantified and normalized against the mature SNORD44.

Pol I transcription is compartmentalized to the nucleolus. Because SNORD44 sdRNAs were much more abundant in the nucleolus than in the nucleoplasm, we asked whether their abundance depends on the functional activity and integrity of the nucleolus. To this end we first treated the cells with actinomycin D (50 ng/ml), which causes abortive rRNA transcription, and assessed the integrity of the nucleolus by nucleolar stress markers NPM and FBL. These showed the segregation of the nucleolus due to Pol I transcription blocks (Fig. 6*A*) [33]. Actinomycin D treatment decreased the abundance of the mature SNORD44, which proportionally led to an increase in the long and sdRNAs (Fig. 6*B*). We then asked whether a decrease in Pol I transcription rate would have a similar impact. For this, we silenced the expression of the Pol I catalytic subunit RPA194 (Fig. 6*C*), or starved the cells for 48 hours by serum deprivation, which led to a decrease in nascent rRNA synthesis as measured by fluorouridine incorporation (Fig. 6*E and F*). In neither case did the treatment affect the level of SNORD44 sdRNAs, nor was the abundance of the long SNORD44 transcript affected in the nuclear fractions (Fig. 6*D and G*). An apparent increase in the long SNORD44 transcript in the cytoplasmic fraction following RPA194 depletion was proportional to the amount of cytoplasmic mature SNORD44 (fold change of mature and long SNORD44 in the control as compared to si-RPA194, 1.3 and 1.1, respectively). These experiments documented that the expression of small and long SNORD44-derived transcripts is independent of Pol I transcription rate, and also suggested that the SNORD44 alternative forms are retained in the nucleolar remnants during nucleolar segregation.

### FBL Binds the Long SNORD44 Transcript and is Required for the Expression of the sdRNAs and the Long RNA Form

**Figure 7:**
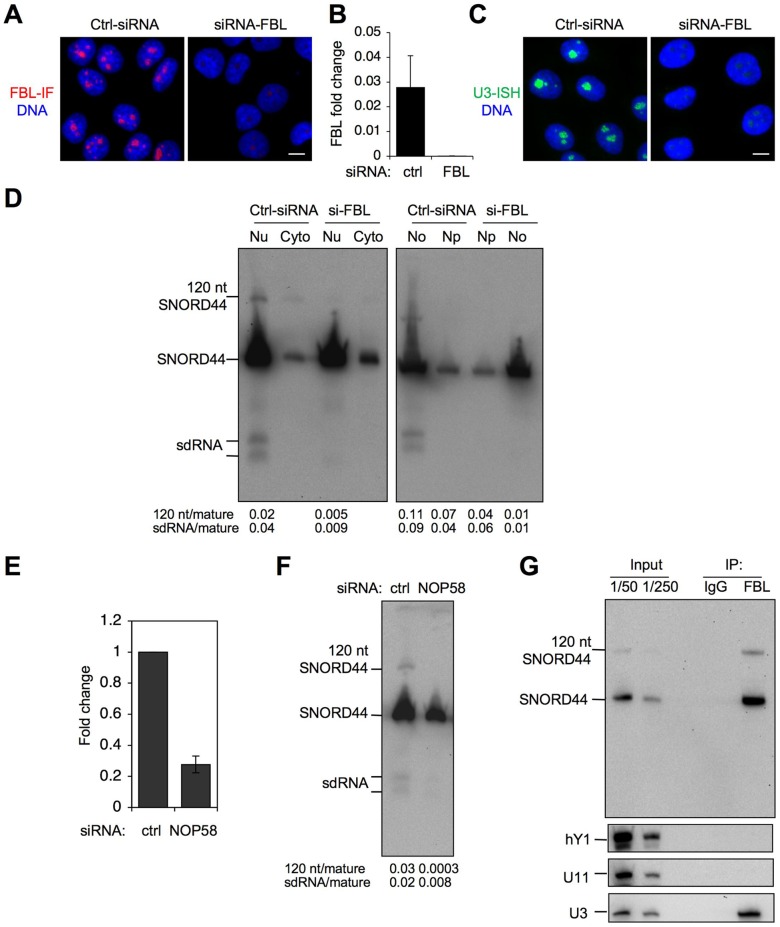
Expression of the SNORD44 sdRNA and long forms depends on FBL and NOP58. (*A–D*) HeLa cells were transfected with control or FBL-targeting siRNAs and incubated for 48 hours. *A* Cells were fixed and stained for FBL and counterstained for DNA. Merged images are shown. Scale bar, 10 µm. *B* Image quantification for FBL. Mean normalized fold intensity is shown. Error bars, SD. *C In situ* hybridization of U3 snoRNA. Scale bar, 10 µm. *D* Nuclear (Nu, containing nucleoli), cytoplasmic (Cyto), nucleoplasmic (Np) and nucleolar (No) fractions were prepared, and RNA was isolated. Northern hybridization was conducted using SNORD44 5′ probe. Signal intensities for 120 nt and sdRNAs were quantified and normalized against the mature SNORD44. (*E, F*) HeLa cells were transfected with control or NOP58-targeting siRNA and incubated for 48 hours. *E* Expression of *NOP58* transcript was determined by qPCR. *F* Northern blotting was conducted for SNORD44 as in D. *G* FBL was immunoprecipitated from HeLa nuclei, followed by isolation of RNA and Northern hybridization using SNORD44, hY1, U3 and U11 probes. IgG was used as negative control in the immunoprecipitation. Inputs represent fraction of RNA present in the nuclei used for the pull-down.

The maturation of snoRNAs and the assembly of snoRNPs depend on the sequential binding of multiple proteins linked to the processing and localization of the complex [8], [39], [45]–[47]. We first tested whether the CRM1-pathway, linked with the nucleolar import of U3 [46], affected the nucleolar expression of the SNORD44 sdRNAs. As analyzed by Northern hybridization, inhibition of CRM1 using leptomycin B modestly decreased the nucleolar abundance of the SNORD44 sdRNAs but not the long form (Fig. S2). We then targeted the box C/D snoRNP component FBL by RNAi. This profoundly depleted the expression of FBL (Fig. 7*A and B*), and also abolished the nucleolar expression of U3 snoRNA (Fig. 7*C*) whose activity is dependent on FBL [39]. As assessed by Northern hybridization and quantification of the RNAs, depletion of FBL strongly reduced the expression of the long SNORD44 transcript and the SNORD44 sdRNAs, while the level of mature SNORD44 was not affected (Fig. 7*D*). Similarly, depletion of NOP58 by siRNA (Fig. 7*E*) decreased the expression of SNORD44 sdRNAs and the long transcript up to 99% and 60%, respectively as compared to the mature SNORD44 (Fig. 7*F*). We then assessed whether the SNORD44 sdRNAs or the long form bind FBL. For this purpose we used FBL immunoprecipitation and analyzed FBL-bound RNAs by Northern blotting using the mature 5′ end SNORD44 probe. The mature SNORD44 and the long form were efficiently co-precipitated with FBL, whereas the SNORD44 sdRNAs were not detected (Fig. 7*G*, *top*), even when exposing the film for a longer time (Fig. S3). As control, U3 RNA co-precipitated with FBL as expected, but the negative control RNAs hY1 or U11 did not (Fig. 7*G*, *bottom*). We conclude that FBL, and possibly other FBL-complex proteins such as NOP58, serves as a factor involved in stabilization, and possibly processing of SNORD44 sdRNAs and the long transcript.

## Discussion

We present here the first analysis of deep sequencing of compartment-specific small RNomes with particular emphasis on nucleolus-associated sRNAs. The deep sequencing of the small RNome revealed unique location-specific features and commonalities. Cytoplasmic and nuclear sRNAs shared similar size-distributions and had high frequencies of miRNA reads, whereas the reads from the nucleolar fraction were dominated by sRNAs mapping to box C/D snoRNAs. The nucleolar sRNA reads had a dual size distribution of 19–20 nt and 25–26 nt as compared to the 22–23 nt reads present in other cellular fractions. Vast majority (98%) of the nucleolar box C/D snoRNA-derived sdRNA reads contained box C, D or both motifs, which was highly suggestive that the motifs were relevant for their processing, localization or both. Conversely, they were largely devoid of guide RNA sequences. This study uncovers a unique small RNome of the nucleolus and demonstrates the expression and regulation of highly abundant sdRNAs from SNORD44 locus.

Our previous analyses of compartment-specific RNAs and proteins show that the cellular fractionation schemes applied here result in effective separation of the cellular compartments and domains [32], [34], [41]. Although it is possible that contamination of the subcellular fractions may comprise some of the analysis of the sdRNAs, several location-specific features in the expression of the sRNAs were detected, including size-distribution and distinct relative abundance of sdRNA reads *e.g.* between the nucleolar and nuclear fractions. In addition, SNORD48, SNORD21 and SCARNA15 were among 14 sRNAs that had higher read numbers in the nuclear compartment than in the nucleolus. This suggests that the sdRNAs may have loci-specific retention or stabilization mechanisms that differ between the nucleolus and the nucleoplasm.

Altogether 68 snoRNAs were represented by 10 reads or more in the dataset. All intronic snoRNA loci located in the *GAS5*, *NOL5* and *SHG1* genes were detected, indicating widespread processing of transcripts arising from these box C/D snoRNA loci. This finding is consistent with the deep sequencing and bioinformatics analyses of cellular sRNAs by Taft *et al*. [24] and Scott *et al*. [27]. Among this wide representation of sdRNAs in the nucleolus, sRNAs mapping to the SNORD44 locus had 20-fold greater abundance than any other sdRNAs. In contrast, only five box H/ACA RNAs (SCARNA15, SNORA48, SNORA64, SNORA73, SNORA8) were present at low abundance according to their sRNA reads in the dataset. Similarly, only few Cajal body RNAs were detected. The nucleolar small RNome thus contained a unique and high content of sRNAs derived from box C/D snoRNAs.

We presumed that rRNA transcriptional activity could affect the expression and localization of the sdRNAs. Following Pol I transcription blocks, the nucleolus undergoes extensive reorganization, including segregation of the subdomains involved in rRNA processing and maturation, and degradation of unassembled rRNAs [48]. The nucleolar abundance of mature SNORD44 decreased in Actinomycin D-treated cells, whereas the long form and sdRNA forms did not. This could indicate destabilization of SNORD44 but not the sdRNA or 120 nt forms. However, the decrease in transcription rate by depletion of the Pol I catalytic subunit or cell starvation, as measured by nascent rRNA synthesis, did not affect the abundance of SNORD44, sdRNAs or its long form. This demonstrates that the nucleolar expression of SNORD44 sdRNAs and long form was independent of Pol I transcription rate, and suggests that the alternative SNORD44 forms were retained in the nucleolar remnants.

Read distribution of the sdRNAs to 5′ and 3′ SNORD ends was diverse. Whereas many sdRNAs aligned with only 5′ ends (*e.g.* SNORD44, SNORD57, SNORD105) or 3′ ends (SNORD2, SNORD27, SNORD66, SNORD78, SNORD100), both 5′ and 3′ end reads of several SNORDs were recorded (SNORD62, SNORD74). Asymmetric processing of snoRNAs and tRNAs has been noted in previous studies [24], [26], [27], [29], [43], [49], and especially for box H/ACA snoRNAs this has been suggested to involve Dicer. However, we did not find evidence of the involvement of either Dicer or Drosha in the generation of the SNORD44 sdRNA fragments. In most mature snoRNAs, the substrate guide region locates near the 3′ terminal box D. We detected nine snoRNA loci (*e.g.* SNORD34, SNORD78, SNORD98) whose sdRNAs contained only the box D, and less than 6% of the sdRNA reads contained a guide RNA sequence. Hence it seems unlikely that the sdRNAs would interfere with the mature snoRNA guide function. However, it is plausible that they could act as decoys for the box C/D binding proteins, especially 15.5K and NOP58, and in such manner limit their availability for snoRNP assembly.

The RNPs that bind snoRNAs are essential for their processing and stability and protect them from exonuclease activity [6], [11], [39]. This was also the case for the SNORD44 sdRNAs. Intriguingly, both FBL and NOP58 were critical for the abundance of the sdRNA and long SNORD44 variants, whereas the mature form was far less affected. The co-regulation of the sdRNA and long SNORD44 forms may suggest that they are coordinately processed. However, no fragment representing the 80–90 nt 3′end of the long transcript was detected by Northern hybridization, suggesting that if the sdRNAs are directly cleaved from the long form, the intermediate 80–90 nt fragment is rapidly degraded. In addition, the binding proteins provide nucleolar localization signals for the snoRNPs, and participate in directing their localizations [50], [51].

## Conclusions

Several studies have suggested that sdRNAs may have miRNA-like properties [22], [23], [26], [30], [31]. If the sdRNAs were to function according to the canonical miRNAs, they should be detectable also in the cytoplasm. However, only a few sdRNAs (deriving from SCARNA15, SNORD44 and U3 loci) were detected in the cytoplasmic fraction. It is possible that the number of sdRNAs is underestimated due to saturation of the cytoplasmic reads by the miRNA, and that there are cell type and physiological variations that affect the expression and localization of individual sdRNAs. Nevertheless, the present data suggests that most sdRNAs are retained in the nucleolar and nuclear fractions, and indicate their functions in other than the typical cytoplasmic silencing of coding genes exerted by the miRNAs. It is noteworthy that similar to Liao *et al*. [25], our study found an almost equally high abundance of cytoplasmic and nuclear miRNAs. It is not entirely conceivable that this should result from contamination of the cellular subfractions, and may thus suggest that mature miRNAs are exposed to constant shuttling between the two compartments. However, this does not appear to be the case for the sdRNAs. In summary, this study adds to the rich representation of small RNAs derived from snoRNAs, and reveals their remarkable nucleolar enrichment. Resolving their nucleolar functions, or whether they merely represent sdRNA degradation products will be needed to understand their potential biological relevance.

## Supporting Information

Figure S1 **Select small RNA reads.**
(TIF)

Figure S2 **Northern analysis of SNORD44 following LMB-treatment.** Cells were treated with leptomycin B (LMB) (10 µM) for 3 hours, fractionated and RNA was isolated. RNA (25 µg) was separated on 15% gel and hybridized to SNORD44 5′ probe. Relative expression of the SNORD44 forms in the nucleolar fraction is shown below.
(TIF)

Figure S3 **Long exposure of Northern hybridization in** **Figure 7F** **.**
(TIF)

Table S1 **Frequency of all snoRNA reads in the subcellular compartments.**
(PDF)
