# Supplementary figures and images for: RNA-Seq of the Nucleolus Reveals Abundant SNORD44-Derived Small RNAs
Source: PLoS One. 2014 Sep 9;9(9):e107519. doi: 10.1371/journal.pone.0107519 (PMC4159348; doi:10.1371/journal.pone.0107519)

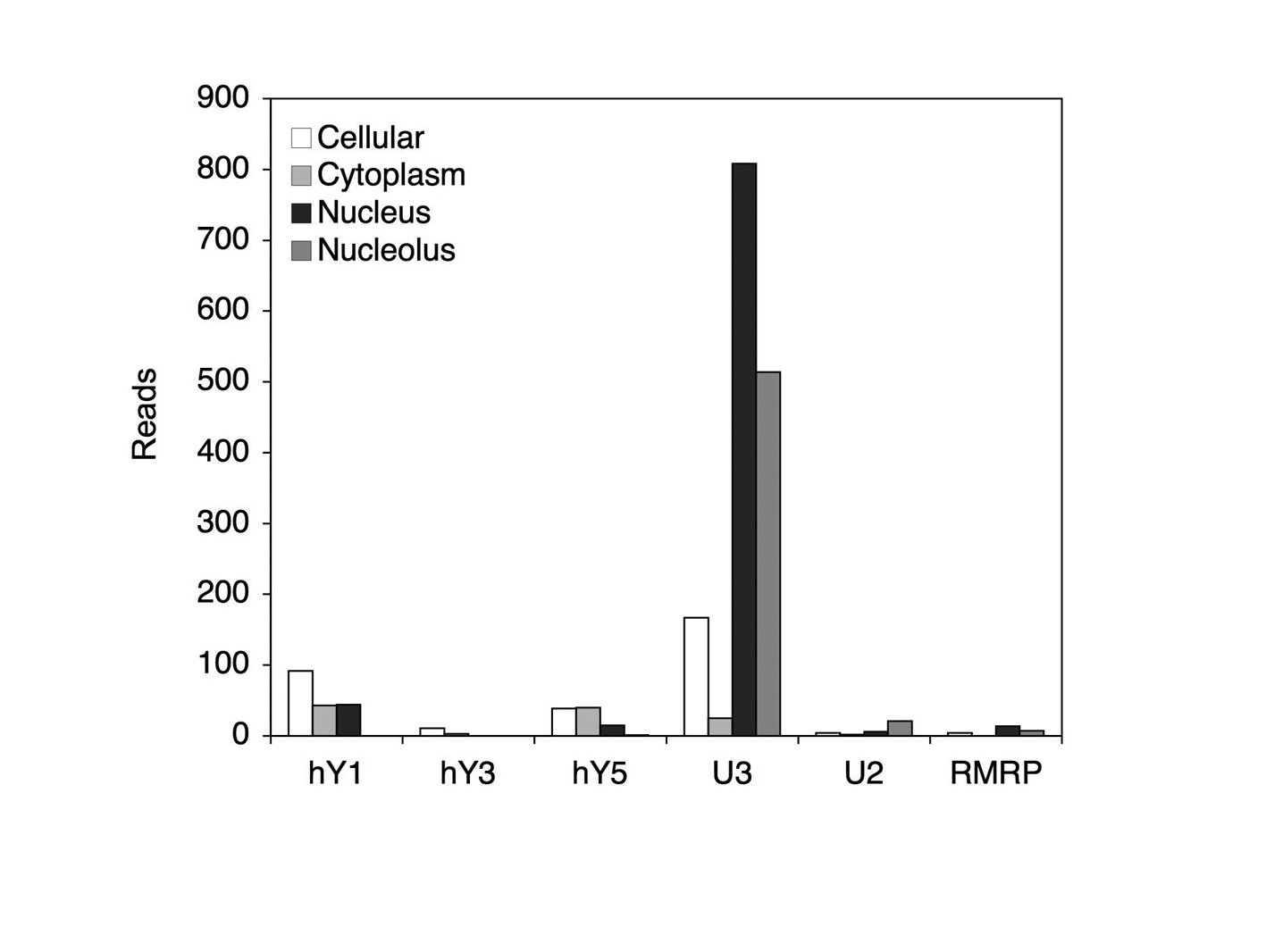

Supplement: Figure S1 — Select small RNA reads. (TIF) [file pone.0107519.s001.tif]

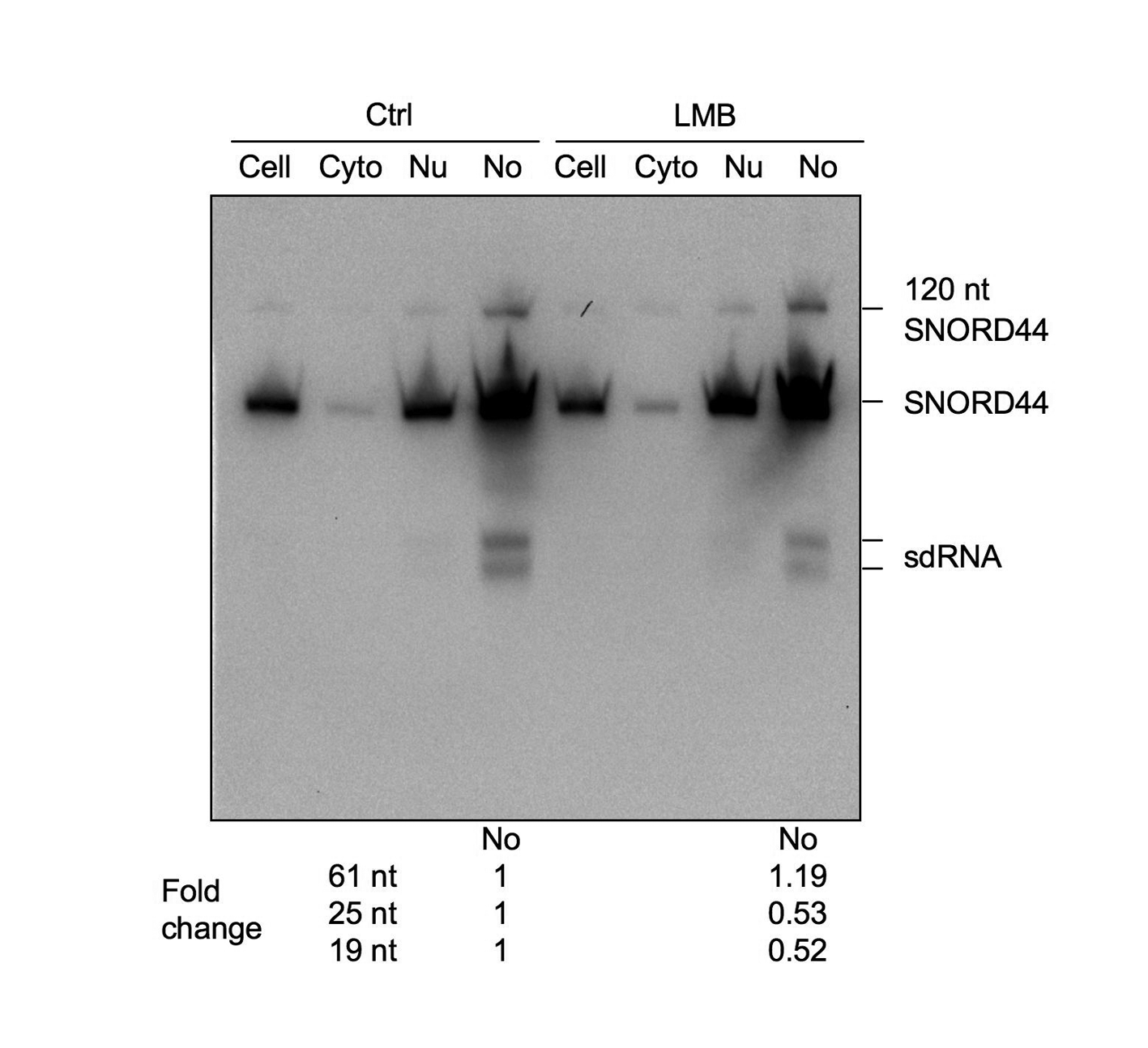

Supplement: Figure S2 — Northern analysis of SNORD44 following LMB-treatment. Cells were treated with leptomycin B (LMB) (10 µM) for 3 hours, fractionated and RNA was isolated. RNA (25 µg) was separated on 15% gel and hybridized to SNORD44 5′ probe. Relative expression of the SNORD44 forms in the nucleolar fraction is shown below. (TIF) [file pone.0107519.s002.tif]

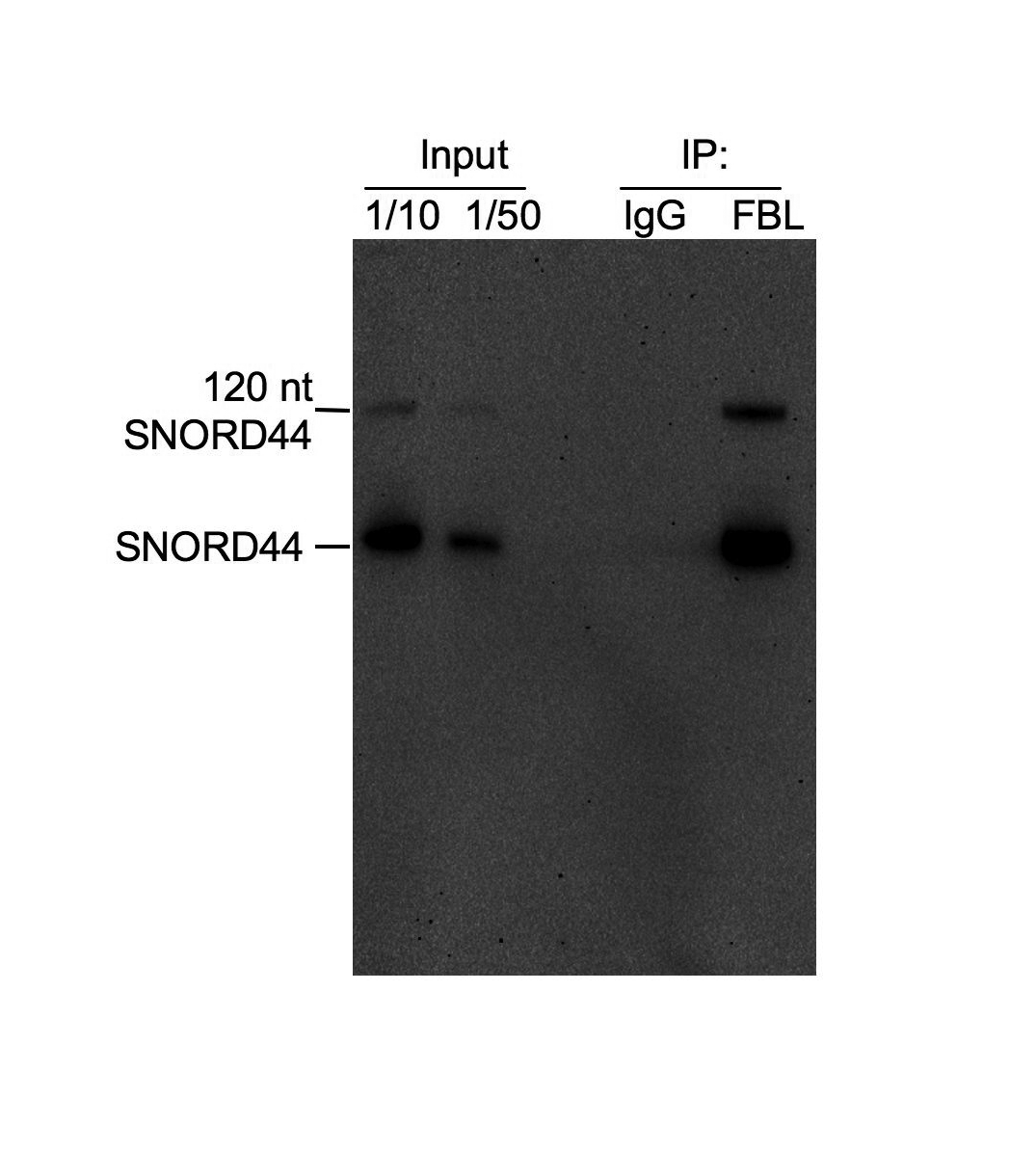

Supplement: Figure S3 — Long exposure of Northern hybridization in Figure 7F . (TIF) [file pone.0107519.s003.tif]
