# Supplementary material for: RNA-Seq of the Nucleolus Reveals Abundant SNORD44-Derived Small RNAs
Source: PLoS One. 2014 Sep 9;9(9):e107519. doi: 10.1371/journal.pone.0107519 (PMC4159348; doi:10.1371/journal.pone.0107519)
Supplement: Table S1 — Frequency of all snoRNA reads in the subcellular compartments. (PDF) [file pone.0107519.s004.pdf]

Table S1. SnoRNA reads identified in the cellular compartments (>10 reads).

Gene	Length	Motif	Target	Reads				% 5' reads				
				Cellular	Cytoplasm	Nucleus	Nucleolus	All	Cellular	Cytoplasm	Nucleus	Nucleolus
SCARNA15	124	H/ACA	U2	28	28	17	0	54.8	64.3	64.3	23.5	0
SCARNA6	266	C/D	U5	4	0	9	20	93.9	75.0	0	88.9	100.0
SCARNA9L2	80	C/D	?	7	1	43	67	89.0	100.0	100.0	88.4	88.1
SNORA48	135	H/ACA	28S	0	0	0	20	100.0	0	0	0	100.0
SNORA64	134	H/ACA	28S	1	0	1	40	0	0	0	0	0
SNORA73	207	H/ACA	?	7	2	34	314	8.1	0	0	5.9	8.6
SNORA8	139	H/ACA	18S,28S	0	0	1	24	100.0	0	0	100.0	100.0
SNORD100	76	C/D	18S	15	1	56	129	6.0	20.0	0	12.5	1.6
SNORD104	70	C/D	28S	5	0	21	79	4.8	0	0	9.5	3.8
SNORD105	85	C/D	18S	3	1	21	253	100.0	100.0	100.0	100.0	100.0
SNORD105B	79	C/D	18S	4	0	12	116	99.2	100.0	0	100.0	99.1
SNORD109	67	C/D	?	0	0	8	18	100.0	0	0	100.0	100.0
SNORD110	75	C/D	28S	2	1	21	26	48.0	100.0	100.0	71.4	23.1
SNORD117	76	C/D	?	0	0	6	10	100.0	0	0	100.0	100.0
SNORD118	136	C/D	28S	2	3	15	4	62.5	50.0	33.3	73.3	50.0
SNORD119	82	C/D	28S	0	0	3	11	100.0	0	0	100.0	100.0
SNORD14C	88	C/D	?	2	0	7	12	57.1	0	0	14.3	91.7
SNORD14E	85	C/D	?	1	0	1	10	100.0	100.0	0	100.0	100.0
SNORD15A	148	C/D	28S	1	0	5	22	96.4	0	0	100.0	100.0
SNORD1A	72	C/D	28S	4	0	8	32	6.8	25.0	0	25.0	0
SNORD1B	84	C/D	28S	2	0	23	0	100.0	100.0	0	100.0	0
SNORD2	70	C/D	28S	37	6	216	177	1.6	8.1	0	1.4	0.6
SNORD20	80	C/D	18S	0	0	5	12	82.4	0	0	100.0	75.0
SNORD21	95	C/D	28S	7	0	36	8	90.2	85.7	0	88.9	100.0
SNORD24	75	C/D	28S	4	0	18	127	8.7	25.0	0	5.6	8.7
SNORD26	75	C/D	28S	2	1	12	110	99.2	100.0	0	100.0	100.0
SNORD27	72	C/D	18S	15	1	91	1777	6.1	40.0	0	39.6	4.1
SNORD3	217	C/D	18S	167	25	808	514	43.7	21.0	16.0	20.9	88.1
SNORD30	70	C/D	28S	10	0	27	52	5.6	10.0	0	11.1	1.9
SNORD31	71	C/D	28S	15	0	59	103	81.4	53.3	0	59.3	98.1
SNORD34	71	C/D	28S	2	0	3	28	3.0	50.0	0	0	0
SNORD36C	68	C/D	28S	6	1	21	3	0.0	0	0	0	0
SNORD38A	70	C/D	28S	7	0	17	39	7.9	0	0	5.9	10.3
SNORD38B	69	C/D	28S	3	0	3	27	33.3	100.0	0	33.3	25.9
SNORD42B	67	C/D	18S	5	0	24	7	11.1	0	0	12.5	14.3
SNORD44	61	C/D	18S	295	27	1496	20414	99.9	99.0	100.0	99.1	100.0
SNORD47	77	C/D	28S	0	0	1	48	100.0	0	0	100.0	100.0
SNORD48	64	C/D	28S	15	0	55	2	56.9	26.7	0	67.3	0
SNORD49A	71	C/D	28S	1	1	10	16	35.7	100.0	0	0	56.3
SNORD4A	72	C/D	18S	0	0	2	15	100.0	0	0	100.0	100.0
SNORD50A	75	C/D	28S	5	0	34	517	98.6	100.0	0	97.1	98.6
SNORD57	72	C/D	18S	6	0	23	216	100.0	100.0	0	100.0	100.0
SNORD58C	64	C/D	28S	0	0	2	10	100.0	0	0	100.0	100.0
SNORD59B	71	C/D	18S	0	0	9	20	89.7	0	0	66.7	100.0
SNORD6	73	C/D	28S	3	0	3	18	20.8	100.0	0	66.7	0
SNORD62	86	C/D	18S	21	2	49	1130	69.6	57.1	50.0	71.4	69.8
SNORD63	68	C/D	28S	2	0	8	25	31.4	0	0	0	44.0
SNORD66	76	C/D	18S	6	1	50	253	1.0	0	0	0	1.2
SNORD68	88	C/D	18S,28S	0	0	0	14	100.0	0	0	0	100.0
SNORD69	77	C/D	28S	2	1	1	19	26.1	50.0	100.0	100.0	15.8
SNORD7	97	C/D	U6	0	0	3	19	95.5	0	0	66.7	100.0
SNORD71	86	C/D	5.8S	3	0	15	107	1.6	0	0	6.7	0.9
SNORD72	80	C/D	28S	1	0	7	64	100.0	100.0	0	100.0	100.0
SNORD74	80	C/D	28S	22	1	106	627	56.9	13.6	0	20.8	64.6
SNORD76	82	C/D	28S	7	0	42	27	88.2	85.7	0	88.1	88.9
SNORD77	68	C/D	28S	3	0	11	19	45.5	0	0	45.5	52.6
SNORD78	65	C/D	28S	83	5	306	344	11.2	15.7	0	20.9	1.7
SNORD80	82	C/D	28S	2	0	7	61	90.0	100.0	0	85.7	90.2
SNORD81	77	C/D	28S	0	1	17	48	4.5	0	0	0	6.3
SNORD82	70	C/D	?	0	0	3	10	30.8	0	0	0	40.0
SNORD83A	95	C/D	28S	0	0	2	11	84.6	0	0	100.0	81.8
SNORD88A	91	C/D	28S	2	0	14	3	100.0	100.0	0	100.0	100.0
SNORD91A	92	C/D	28S	4	0	25	178	0.5	0	0	4.0	0
SNORD91B	95	C/D	28S	1	1	12	50	6.3	0	0	25.0	2.0
SNORD93	74	C/D	18S	5	0	17	10	62.5	100.0	0	76.5	20.0
SNORD96A	76	C/D	5.8S	0	0	3	74	97.4	0	0	33.3	100.0
SNORD97	142	C/D	?	7	0	11	7	48.0	14.3	0	36.4	100.0
SNORD98	67	C/D	18S	1	1	30	27	3.4	0	0	6.7	0
				865	112	4026	28594	56.7	36.6	9.8	52.6	54.8
